# Mapping Psychosocial Interventions for Psychosis and Schizophrenia Across Gulf Countries: A Scoping and Narrative Review

**DOI:** 10.3390/jcm15135103

**Published:** 2026-06-30

**Authors:** Zahra Khalesi, Maisara Sukar, Noor Sharif, Natalie Tayim

**Affiliations:** Clinical Psychology Program, School of Social Sciences and Humanities, Doha Institute for Graduate Studies, Doha P.O. Box 200592, Qatar; msu002@dohainstitute.edu.qa (M.S.);

**Keywords:** schizophrenia, psychosis, psychosocial interventions, Gulf Cooperation Council, scoping review

## Abstract

**Background/Objectives:** Interest in mental health research from the Arab world has grown in recent years, yet evidence on effective care remains uneven across subregions. The unique landscape of the Gulf countries underscores the need for culturally responsive psychosocial interventions for these vulnerable populations. This scoping review aimed to map psychosocial interventions for psychosis and schizophrenia that have been evaluated in Gulf countries, including Bahrain, Kuwait, Oman, Qatar, Saudi Arabia, and the United Arab Emirates. **Methods:** Following scoping review methodology and PRISMA-ScR reporting guidance, we conducted systematic searches across five databases (APA PsycINFO, PubMed, Embase, Scopus, and Web of Science). Records were screened using predefined eligibility criteria and grouped thematically based on intervention type. **Results:** Ten studies met the inclusion criteria, including six English-language studies and four Arabic-language studies. Studies were conducted in Saudi Arabia, Kuwait, Oman, and the United Arab Emirates, with no eligible studies found from Bahrain or Qatar. Intervention types clustered into four categories: psychosocial rehabilitation platforms, social and functional skills interventions, caregiver-focused psychosocial interventions, and transdiagnostic studies. Outcomes, cultural adaptation processes, and methodological reporting varied considerably across studies. **Conclusions:** The findings highlight gaps in intervention development, evaluation standards, and the reporting of cultural adaptation, which may inform culturally responsive service planning for people with psychosis and schizophrenia across the region. Future studies should use standardized concepts, validated outcome measures, and clearer reporting of cultural adaptation processes to support direct comparisons and improve treatment evaluation across Gulf countries.

## 1. Introduction

Difficulty distinguishing reality from non-reality has been ubiquitously documented and categorized within psychotic experiences [1]. Clinically, there is a broad understanding that psychotic experiences may not be unique to psychotic disorders but rather may manifest as transient responses to various stressors that may be related to a combination of an individual’s developmental background, genetics, substance use, and current stressors [2]. Due to the significant heterogeneity in presentation, prognosis, and impact, most clinical practices have begun to shift away from the diagnostic labels of psychotic disorders (such as schizophrenia, schizoaffective disorder, delusional disorder, and brief psychotic disorder [3,4]) and instead focus on the transient experience of psychosis for the individual [5,6]. Nevertheless, the majority of academic literature continues to use standardized classification systems, such as the International Classification of Diseases (ICD) or the Diagnostic and Statistical Manual (DSM) [7], and their related diagnostic labels. The construct of interest is the clinical experience or phenomenological symptom cluster of hallucinations, delusions, and disorganized thinking [8,9]. Within the schizophrenia disorders lens, there are additional cognitive and negative symptom domains, as well as functional impairment that is inherent to the diagnostic label, thereby indicating greater intensity [10]. To help circumvent the discrepancy in language use arising from regional differences, this review will focus on psychotic experiences broadly through the use of diagnostic labels such as schizophrenia or schizophrenia spectrum disorders in order to maximize relevant search results.

Psychotic experiences are among the most heterogeneously reported experiences [11]. That is, while symptom clusters exist, no two individuals with psychosis (or schizophrenia) share the same story. These differing experiences may be related to a combination of individual difference factors [12,13], attributions of the symptoms [14,15,16], cultural influences [17,18,19], and medical influences relating to health and genetics [20,21]. Here we see that while medical attributions contribute to some of the differences observed, the overall environmental context of the individual impacts their ability to respond, interpret, and integrate knowledge. This concept, often referred to as meaning-making [22,23], is integral to our general understanding of ourselves [23] and to understanding the impact of a psychotic episode [22]. When individuals encounter psychotic experiences, they assign an “appraised meaning” to these situations; the extent to which this appraisal is discrepant with their global meaning determines the level of distress they experience [23]. For instance, ethnic groups with higher spiritual attributions often demonstrate more benign symptom profiles and improved outcomes [24], suggesting that such frameworks may facilitate a reduction in discrepancy-related distress.

The cultural context also shapes the phenomenological profile of the disorder. While visual hallucinations are more common in African, Asian, Middle Eastern, and Caribbean patients [25], auditory hallucinations are significantly more prevalent in Western cultures, with reports indicating a prevalence of 83.4% in US samples compared to 64.3% in other regions such as India [26]. Beyond frequency, the appraisal of these symptoms is often marked by ambivalence. According to the Meaning-Making Model [23], local cultural contexts and specific causal attributions—such as spiritual versus biomedical explanations—function as “global meaning” or orienting systems that provide individuals with cognitive frameworks to interpret their experiences. While negative appraisals generally prevail, many patients experience mixed attitudes or even predominantly positive associations, such as perceiving voices as social surrogates or companions [27,28]. This complexity is further reflected in the defensive role of persecutory ideas; positive symptoms may serve an implicit purpose in maintaining self-esteem and decreasing discrepancies between the “ideal” and “actual” selves through an external-personal attributional style [29]. Ultimately, the process of meaning-making is central to personal recovery, which is understood as finding self-coherence and a sense of personal dignity beyond simple symptom reduction [29]. Recovery involves a deeply subjective restoration of a meaningful life and the ability to make sense of the challenges posed by serious mental illness, effectively reducing the discrepancy between the experience of illness and one’s global goals to restore a sense of worth [23]. Because meaning-making processes are inherently shaped by cultural context, they should serve as a primary informant for the cultural adaptation of psychosocial interventions globally [30,31], providing the logical foundation for translating subjective recovery experiences [29] into systematic clinical frameworks [23].

Cultural adaptation of psychosocial interventions has therefore become a recurring theme in the global mental health literature [30,31,32]. Indeed specific frameworks have been developed in order to ensure the relevance and efficacy of the adaptations for particular contexts including: modifying intervention content, changing the context (where and by whom an intervention is delivered) and considering fidelity to the original intervention [31]. Such adaptation strategies have been previously documented within the psychosis and schizophrenia literature, ranging from integrating traditional healers and addressing structural poverty in sub-Saharan Africa [33,34] to aligning with family decision-making in East and South-East Asia [35], to attending to migration and language in European host countries [36]. Ultimately, synthesizing these culturally tailored approaches is essential to bridging the gap between universal biomedical frameworks and the specific local realities of patients worldwide. These studies have ultimately demonstrated the necessity of integrating cultural beliefs in psychosocial intervention responses while underscoring the idiosyncratic needs of the individuals within their respective cultures. That said, the direct transferability of the adaptation strategies mentioned above does not take into account the heterogeneous cultures that must co-exist within one geographical setting.

Indeed, the unique landscape of complex socio-demographic populations such as those seen in the Gulf Cooperation Council countries (GCC; Bahrain, Kuwait, Oman, Qatar, Saudi Arabia, and the United Arab Emirates) exemplifies the nuanced needs of the region. Since the mid-20th century, the GCC has seen a massive influx of international migrants, leading to foreign residents outnumbering nationals in several states [37,38]. This rapid shift has created a society defined by a unique “demographic imbalance,” where diverse ideas regarding faith, social shame, and mental health responses coexist in a highly segmented environment. The speed of this modernization meant that these diverse cultural narratives were often layered upon one another without sufficient time for social integration [39], and consequent erosions of traditional, rural societal customs and structures were reported [40]. Of note, Qatar, Saudi Arabia, and the United Arab Emirates now balance ultra-modern metropoles while upholding Islamic cultural, social, and legal rules [39,41,42,43]. The coexistence of vastly differing cultures can lead to frameworks that may not be applicable to some clients. For example, over one-third of women in polygamous marriages in Kuwait attribute their illness to complicated marriage dynamics [39]. As a result, mental health providers must now navigate a complex landscape where traditional attributions for psychosis, such as religious or spiritual explanations, intersect with modern biomedical interventions in a population that has had to adapt to these changes in real-time [39]. A practical consequence for clinicians is a complex landscape where traditional attributions for psychosis and schizophrenia intersect with biomedical interventions in a population that has had to adapt to these changes in real-time and where mismatches between provider and patient frameworks affect engagement and treatment adherence.

Approximately 10 years ago, a scoping review of mental health research in the Gulf region identified studies on cultural variations in symptom presentations and early initiatives to address these differences [39]. Since then, studies have continued to highlight demographic and presentation-related differences in patients with schizophrenia, as well as the need for culturally adapted interventions [40,44,45,46,47,48]. For instance, studies from Oman have identified relatively young individuals with psychosis symptoms alongside low service utilization [40]. Clients who sought traditional healers first experienced a significantly longer duration of untreated psychosis [40], which is a known risk factor for poor prognosis [49,50]. Family involvement and beliefs contributed to delayed or refused treatment, often through denial of mental disorders and adherence to superstitious explanations such as demonic possession [40,46]. Specifically, attributions of possession or witchcraft significantly delayed treatment. In Saudi Arabia, clients tend to express physical rather than mental health symptoms due to high community stigma [51]. Further studies on Saudi Arabian nationals found that nearly 60% of patients experienced high levels of societal stigma, internalized stigma, and associated feelings of isolation [45]. Research on family and extended family involvement has revealed substantial caregiver burden related to physical and mental strain, stigma, violence, and unmet needs [44,46,47,52,53,54]. Furthermore, differences in literacy and socioeconomic status have been reported but not yet addressed in psychosocial interventions [47,48]. The studies published in this decade highlight the specific needs of this region, while continuously urging researchers to incorporate such information into translational, intervention studies in the implications and future directions sections. Alongside this growing need, regional governments and health systems have continued to expand mental health services and funding in hospital and community settings [38,55,56,57,58].

This scoping review therefore aims to achieve the following objectives: (1) map the psychosocial interventions for psychosis and schizophrenia that have been evaluated in Gulf countries, (2) characterize their study designs, populations, and outcomes, (3) describe how these interventions have been implemented, and (4) identify whether and how cultural adaptation has been reported. The findings are intended to clarify the evidence base, identify priorities for future evaluation, and inform clinical practice and service development for culturally responsive care across the region.

## 2. Materials and Methods

### 2.1. Design and Reporting Framework

This scoping review was guided by the JBI methodology for scoping reviews [31], and is reported in accordance with the PRISMA-ScR reporting guidance [32]. A protocol was developed for the review but was not registered (see Supplementary File S1).

### 2.2. Review Question

What psychosocial interventions for psychosis and schizophrenia have been evaluated in the GCC countries, and to what extent have cultural adaptations been reported?

### 2.3. Eligibility Criteria

Studies were eligible if they: (1) evaluated a non-pharmacological psychosocial intervention or service model delivered to people with psychosis or schizophrenia, or to their family members or caregivers; (2) were conducted in a Gulf country (Bahrain, Kuwait, Oman, Qatar, Saudi Arabia, or the United Arab Emirates); and (3) reported an evaluation component with outcomes (e.g., symptoms, relapse or readmission, functioning, quality of life, caregiver outcomes, social or functional skills, or service use and cost outcomes). We included a broad range of quantitative evaluation designs (e.g., randomized or non-randomized controlled studies, quasi-experimental studies, pre-post evaluations, pilot studies, and service or program evaluations). We also included case reports or case series in which a psychosocial intervention was delivered and outcomes were described. We excluded medication-only studies, purely descriptive or observational studies with no intervention evaluation, scale development or validation studies, and qualitative-only studies without an intervention evaluation component. English- and Arabic-language publications were eligible, including peer-reviewed articles and relevant gray literature (e.g., dissertations) where the full text was available and met the inclusion criteria. No date limits were applied.

### 2.4. Information Sources and Search Strategy

Since both English and Arabic publications were eligible for inclusion, the search strategy was designed to identify relevant literature across international bibliographic databases and Arabic sources. Systematic searches were conducted on 26 January 2026, in five databases: APA PsycINFO, PubMed, Embase, Scopus, and Web of Science, from database inception to the search date. Search strategies combined three concepts: psychosis or schizophrenia, psychosocial interventions, and Gulf countries. The English search strategy was developed first and adapted for each platform using database-specific syntax, field tags, and controlled vocabulary where available. Arabic-language studies were identified through targeted searches in three Arabic databases: Dar Almandumah, Al Manhal, and e-Marefa. These searches used equivalent Arabic terms for schizophrenia or psychosis, psychosocial interventions, and Gulf countries. Because search functions varied across Arabic database interfaces, Arabic-language records were documented as additional sources rather than as fully reproducible database searches. However, all Arabic records were screened using the same predefined eligibility criteria as the other database results. Example search terms are shown in Table 1, and full search strategies for the English-language databases are provided in Supplementary File S2.

### 2.5. Selection of Sources of Evidence

All records were imported into Rayyan for management and deduplication (Rayyan Systems Inc., Cambridge, MA, USA; web application accessed in January 2026) [59]. Title and abstract screening were completed using the predefined eligibility criteria. To assess screening consistency at the title and abstract stage, a second reviewer independently screened a 15% sample of records in a blind mode. Full-text screening was conducted independently by two reviewers, and disagreements were resolved at the level of the research team through documentation and discussion as per the JBI guidelines [60]. 

### 2.6. Data Charting Process and Data Items

Data extraction and charting were conducted using a structured template. Extracted items included citation details, country and setting, study aims, design, sample and diagnosis, intervention components and delivery characteristics, comparator, outcomes and measures, timepoints, key results, and limitations. The data charting framework and extraction fields were developed and agreed upon by two reviewers prior to extraction, then piloted on an initial subset of studies and refined before full data extraction in accordance with recommendations for scoping reviews [60]. One reviewer completed data extraction for all included studies. Arabic-language full texts were screened and data were extracted by an Arabic-fluent reviewer and translator, and charted using the same framework as the English-language studies. Disagreement arose on two out of 110 records reviewed in “blind mode”, and on one of the eight studies during the full-text review. The two reviewers discussed disagreements with reference to the inclusion and exclusion criteria until consensus was reached. All disagreements and resolutions were documented in Rayyan. An adjudicator was available on standby for any unresolved conflicts but was not needed.

### 2.7. Critical Appraisal

Consistent with the aims of this scoping review, a critical appraisal of individual sources of evidence was not performed.

### 2.8. Synthesis of Results

Findings were synthesized descriptively and narratively. Extracted data were first charted by citation details, country, setting, study design, sample and diagnosis, intervention components, comparator, outcome domains, key findings, and the reporting of cultural adaptation. Studies were then grouped into four intervention categories according to the intervention target, delivery setting, and primary outcome domain. These categories were discussed among the authors and refined until consensus was reached. The narrative synthesis was organized around these intervention categories and interpreted in relation to gaps in the regional evidence base and cultural adaptation. Due to the small number of studies identified, results are presented with a detailed narrative summary within each category. Diagnostic labels were kept in line with each publication’s descriptions despite the use of nomenclatures that are no longer standard. This is particularly relevant for studies that use “paranoid schizophrenia” or “paranoid type” as represented by the older included studies [61,62].

### 2.9. Reflexivity

The co-authors of this manuscript are bilingual, are of Arab heritage, and are affiliated with a university in Qatar. These shared linguistic and cultural backgrounds may have facilitated a deeper sensitivity to contextual and cultural nuances within the studies reviewed, particularly where such nuances were not explicitly articulated. This positionality likely enhanced our ability to interpret meaning embedded in language, social norms, and culturally specific constructs.

At the same time, we recognize that this proximity to the cultural context may have introduced interpretive bias, including the potential to over-attribute meaning or infer intentions beyond what was explicitly reported in the data. We remained attentive to this possibility throughout the analytic process by grounding interpretations in the available evidence and engaging in ongoing critical reflection and discussion among the co-authors.

## 3. Results

**Figure 1:**
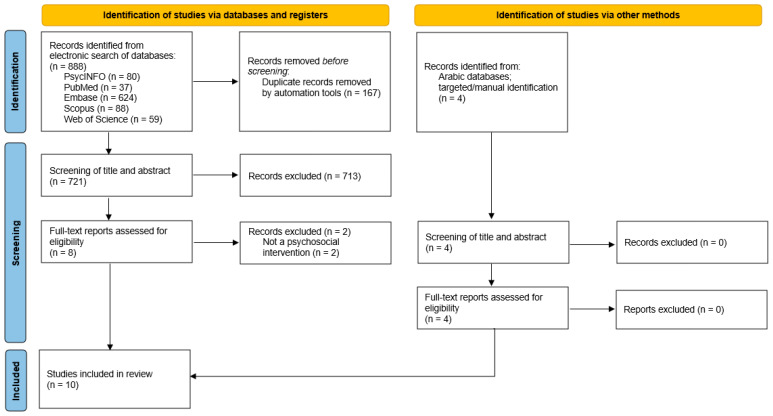
PRISMA flowchart of studies identified for inclusion in scoping review about psychosocial interventions evaluated in the Gulf.

Initial search results identified 888 records, of which 167 were removed during deduplication. Overall, 98.8% of records were excluded based on the eligibility criteria described above, and four Arabic-language studies were included from other search strategies (see Figure 1 for more detail).

A final ten studies met the inclusion criteria and were mapped across four Gulf countries, with most evidence originating from Saudi Arabia and Kuwait. The included evidence clustered into four intervention categories: psychosocial rehabilitation platforms, social and functional skills interventions, caregiver-focused psychosocial interventions, and transdiagnostic studies. Study designs were heterogeneous and mostly non-randomized, ranging from small controlled studies and quasi-experimental designs to pre-post program and service evaluations. Several included studies were Arabic-language intervention evaluations, and one included source was a doctoral dissertation. Outcomes varied by intervention category and included symptom measures, quality of life and functioning outcomes, service utilization and cost outcomes, caregiver outcomes, and social skills outcomes.

Table 2 outlines the general characteristics of the included studies. Participant diagnoses or target diagnostic populations were reported across all included studies. Six studies focused specifically on schizophrenia spectrum disorder populations, including patients with schizophrenia, chronic schizophrenia inpatients, recovered schizophrenia participants, or caregivers of people with schizophrenia.

Building on the general study characteristics shown in Table 2, Table 3 summarizes the intervention characteristics, delivery format, comparator conditions, and the reporting of cultural adaptation or local context across the included studies.

**Figure 2:**
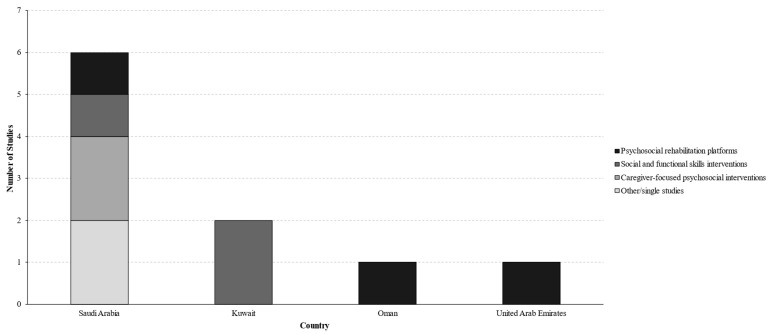
Intervention categories by country.

**Figure 3:**
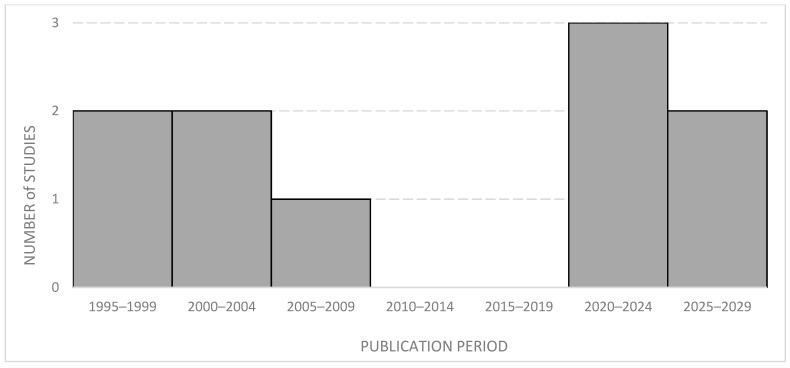
Included studies by publication period.

After summarizing the study and intervention characteristics, we mapped the included studies by country, intervention category, and publication period. Figure 2 displays the intervention categories by country, and Figure 3 displays the number of included studies by publication period. The oldest included study was published in 1995 and the most recent studies were published in 2025. There were no studies that met the inclusion criteria published between 2008 and 2021, highlighting a 13-year gap in published findings regarding psychosocial interventions for patients with psychosis or schizophrenia in the Gulf. The country with the most publications was Saudi Arabia (*n* = 6), accounting for 60% of the included studies and demonstrating the greatest diversity of intervention types. Kuwait had the second largest output of studies (*n* = 2), followed by Oman (*n* = 1), and the United Arab Emirates (*n* = 1). Three countries, Saudi Arabia, Oman, and the United Arab Emirates, published findings on psychosocial rehabilitation platforms or general psychiatric service models. Kuwait and Saudi Arabia were the only countries to report social and functional skills interventions for schizophrenia-related populations. No intervention evaluations were identified from Qatar or Bahrain in the included full-text set.

Study designs varied considerably and were mostly non-randomized. Participant diagnoses or target diagnostic populations were reported in all ten studies, although the diagnostic classification system was not clearly reported in eight studies [62,63,64,65,66,67,69,70]. Of the two studies that reported diagnostic classification procedures, one used ICD-10 criteria for schizophrenia [61], and one used DSM-IV criteria with the SCID-I [68]. Designs included controlled intervention studies with comparison groups, one single-group pre-post-follow-up intervention study, comparative or pre-post service evaluations, and one exploratory dissertation case series. Sample sizes ranged from 3 to 354 participants. Target recipients included inpatients in 30% of studies [61,62,64], outpatients in 10% of studies [68], day treatment patients in 20% of studies [63,68], and caregivers of patients with schizophrenia in 20% of studies [67,70]. Four studies included transdiagnostic psychiatric samples with approximately 33.0% of participants diagnosed with schizophrenia or paranoid schizophrenia [62,63,66,68], and one study recruited patients with chronic schizophrenia but did not clearly specify their inpatient/outpatient status [65].

Sex distribution was not consistently reported by diagnosis across studies involving patients with schizophrenia or schizophrenia-related populations. Among the two caregiver-focused studies, 40% of caregiver participants were explicitly identified as mothers [67,70].

As shown in Table 2, the majority of publications were in English (60%), while four studies were published in Arabic (40%): two from Saudi Arabia and two from Kuwait. Psychology or psychological/behavioral sciences authors acted as the first author on half of the included publications (50%), followed by psychiatry or mental health service leadership (20%). First-author backgrounds also included psychiatric and mental health nursing (10%) and art education (10%). The first-author discipline was not clearly specified in one case (10%), and one of the included studies was gray literature: a doctoral dissertation in art education from The Pennsylvania State University, School of Visual Arts, with fieldwork conducted in Saudi Arabia [62].

### 3.1. Evidence Map: Intervention Categories Identified

Following the descriptive mapping of study characteristics, interventions were grouped into four categories according to the intervention target, delivery setting, and primary outcome domain. These categories comprised psychosocial rehabilitation platforms, social and functional skills interventions, caregiver-focused psychosocial interventions, and transdiagnostic studies. Categories are presented from highest to lowest frequency.

(1) Psychosocial rehabilitation platforms

Three studies evaluated psychosocial rehabilitation platforms that delivered care beyond routine outpatient visits [63,66,68]. Of those, two were day treatment models [63,68] and one was a community outreach service [66]. Both day-treatment studies included transdiagnostic psychiatric samples, with schizophrenia representing approximately one-third of each sample; however, only the United Arab Emirates study reported schizophrenia-specific outcomes [68]. These studies described a general day treatment model with several planned activities, such as medication management, social support, rehabilitation, occupational individual and group sessions, physical training, computer classes and games, painting, and handicrafts, though they did not detail whether any specific adaptations were made for the patients [63,68]. In one study, schizophrenia-specific outcomes revealed improved cognitive and social functioning for those who engaged in day treatment [68], while the other day treatment study reported overall rehabilitation improvement for the full engaged sample, without reporting outcomes separately by diagnostic category [63]. The final study evaluated a community outreach service (community mental health service; CMHS) for patients with schizophrenia in Oman [66]. Reported activities primarily focused on medication management and support, including assessment, medication provision or administration, case management, blood investigations, and multidisciplinary team follow-up. Although the authors identified significant decreases in relapse and future hospitalization stays [66], the multicomponent nature of the service makes it difficult to isolate the psychosocial contribution from medication engagement, case management, and community follow-up.

(2) Social and functional skills interventions

Three studies evaluated interventions targeting social or functional recovery domains among schizophrenia-related populations, all of which were published in Arabic [64,65,69]. Study methods and outcomes varied considerably, and therefore direct comparisons or integrations could not be made. Two of the studies described the implementation of adapted CBT-based interventions aimed at improving communication skills [65] or addressing “social phobia” [69]. Specific details on the content of delivery or the ways in which the sessions were adapted to reflect local needs were not provided. Delivery frequency differed considerably, with one implementing a high-frequency intervention delivered twice a week over 4 weeks for 90 to 120 min per session [65], while the other was delivered as a single, four-hour session with breaks [69]. Finally, the third study employed a token-based behavioral program to reward socially desired behaviors for inpatients with schizophrenia within an inpatient setting. Details on the methodology or rigor were not provided, and the findings demonstrated an increase in the desired behaviors that were explicitly rewarded by the inpatient staff [64].

(3) Caregiver-focused psychosocial interventions

Two studies evaluated psychosocial interventions targeting caregivers of people with schizophrenia [67,70], one of which was published in Arabic [67]. Although both studies identified a need for a psychoeducation program for caregivers of people with schizophrenia in Saudi Arabia, they highlighted different domains and frequency of interventions, with one operating daily over a four-week period through a virtual network, while the other encompassed 14 sessions delivered three times per week [67]. Sharif and colleagues [70] highlighted schizophrenia psychoeducation, treatment and medication adherence, communication and home safety, risk assessment, the management of delusions and hallucinations, suicide prevention, stigma, family support, caregiver needs, and community services in their intervention. They found a significant decrease in family burden, as measured by the Family Burden Interview Schedule, among participants following the intervention. In contrast, Al Rashoud [67] focused on concepts of psychological hardiness, self-confidence, supporting the expression and discussion of thoughts and feelings, improving morale and social interaction, supporting the acceptance of the caregiving situation, and helping mothers identify goals and responsibilities. They reported significant changes using a measure constructed by the authors for the study and not previously validated, raising concerns about potential bias.

(4) Transdiagnostic studies

Two of the studies evaluated were published approximately 30 years ago [61,62], one of which was a doctoral dissertation that evaluated a transdiagnostic expressive therapy and included a case of paranoid schizophrenia [62]. Both studies used culturally relevant elements in delivery (Islamic prayer; Saudi tradition framing; Arabic-language delivery; locally developed tools) but did not document an adaptation process or framework. The intervention for voices included recommendations such as the structured use of prayer, Quran reading, Islamic guidance audio, and the doctrinal reframing of religious or superstitious voice content, but it did not specify how these components were implemented or integrated with cognitive-behavioral therapy [61]. The other study evaluated a transdiagnostic expressive therapy that included a paranoid schizophrenia case at King Khalid University Hospital in Riyadh [62]. Graphic reenactment was described as an interactive art therapy technique in which the therapist used art to mirror or respond empathically to the patient’s feelings and thoughts, open communication, and offer therapeutic direction, with verbal communication limited but not eliminated. The dissertation broadly situated art therapy within Saudi Arabian cultural traditions, values, and Islam, but did not describe a manualized component. The outcomes were confounded by situational experiences for the patient, and confident conclusions about the efficacy of this intervention could not be drawn [62].

### 3.2. Outcomes Assessed Across Studies

Consistent with the scoping aim of mapping what has been evaluated rather than pooling effects, outcomes varied considerably across studies and no standardized outcome measure was used across more than one included study (see Table 4 for details). Psychosis symptom-specific outcomes were explicitly measured in only one study using the Structured Auditory Hallucinations Interview (SAHI) and a locally developed symptom scale [61]. Within each intervention theme, the outcomes reported varied considerably, making direct comparisons difficult to ascertain. For example, service utilization and cost outcomes, including admissions/readmissions, length of stay, and costs, were prominent in the community mental health service evaluation from Oman [66], while quality of life and functioning outcomes were central to the day treatment studies from the United Arab Emirates and Saudi Arabia [63,68]. Within the caregiver-focused interventions, the primary outcomes reported were conceptually distinct: caregiver burden, measured using the Family Burden Interview Schedule [70], and psychological hardiness, measured using a researcher-developed scale [67]. Finally, social and functional outcome studies also reported conceptually distinct tools, including measures emphasizing conversation skills, self-assertion, communication, and self-care [64,65], while another broadly defined social phobia [69]. Overall, the degree of detail reported, choice of measures, and outcome constructs differed across all ten studies, highlighting a large discrepancy among studies in the GCC.

### 3.3. Reporting of Cultural Adaptation

The studies included in this review often acknowledged the relevance of cultural context through the use of culturally relevant elements, though they rarely documented a cultural adaptation process. Only two studies explicitly discussed the cultural grounding of the interventions [61,62], while most studies did not describe a formal process for adapting intervention content, delivery, or therapeutic techniques. Of the two studies that discussed cultural grounding, neither of them documented a specific adaptation framework or described procedures for documenting the relationship between original intervention content, adapted content, cultural context, and fidelity, as recommended in published adaptation guidance [30,31,32]. Wahass and Kent [61] operationalized adaptation most clearly by incorporating Islamic practices and doctrinal reframing into intervention components for persistent auditory hallucinations, including the structured use of prayer, Quran reading, Islamic guidance audio, and religiously grounded reframing of threatening or superstitious voice content, though little information was provided on how those recommendations were aligned with the treatment framework in order to ensure fidelity and efficacy. Alyamy (1995) [62] also situated graphic reenactment art therapy within Saudi Arabia’s cultural traditions, values, and Islam, but briefly framed this more as cultural grounding for the intervention without detailing an adaptation process or framework model. Other studies reflected local context through Arabic-language publication or implementation [64,65,67,69], locally developed or adapted measures [63,64,65,67,69], and, where reported, translation or back-translation processes [63,68]. However, these studies did not describe a process for culturally adapting intervention content, delivery, or therapeutic techniques beyond language, measurement, or service-context modifications. These studies depict the use of superficial, culturally relevant additions, rather than an integrated cultural adaptation framework to determine how culture shaped the intervention, whether adaptations were systematic, or whether adaptations influenced acceptability, fidelity, and outcomes.

### 3.4. Reproducibility of the Research

Meaningful comparisons or systematic assessments of methodological quality were difficult to make across studies due to the large variation in methodological tools and interventions. Four studies (40%, all Arabic-language studies) used researcher-developed, modified, or study-specific measures [64,65,67,69] that did not report psychometric validity in the target sample. The use of such instruments raises concerns about bias, reliability, and validity. When existing tools or interventions were translated, modified, or culturally contextualized, the authors frequently did not describe the adaptation process in sufficient detail to determine how changes were tailored to the local cultural context or how fidelity was maintained [61,62,63,68]. Only one study clearly reported Arabic validity/reliability evidence for the primary outcome measure [70], while others used modified tools without consistently establishing psychometric validation for the target sample and setting [61,62,63,68]. No standardized outcome measure was used across more than one included study. The evidence base was further constrained by generally small sample sizes [61,62,64,65,69], and by a notable absence of replication studies, limiting confidence in the robustness and generalizability of the findings. Moreover, the methodology sections often lacked procedure or adaptation details, had limited demographic information, and used inconsistent measures [64,65,67,69]. Additionally, the absence of randomized controlled trials across the included literature significantly restricts the strength of causal inferences that can be drawn.

## 4. Discussion

The current scoping review highlights a significant geographic and methodological imbalance in the research landscape of psychosocial interventions for psychosis across Gulf countries. While ten studies were identified across four countries, the evidence is heavily concentrated in Saudi Arabia (60%) and Kuwait (20%), leaving substantial gaps in our understanding of effective practices in nations like Bahrain and Qatar, where no eligible studies were identified despite documented needs [71,72]. The identified interventions primarily cluster into four categories: psychosocial rehabilitation platforms, social and functional skills interventions, caregiver-focused psychosocial interventions, and transdiagnostic studies. The utility of these findings is constrained by a high degree of heterogeneity in study designs, which predominantly rely on non-randomized, small-scale evaluations [65,67]. Methodological critiques in this review focus on specific features such as the use of unvalidated, locally developed measures and limited demographic reporting [64,67]. For instance, several Arabic studies employed researcher-developed scales without reporting established validity in the target population, which restricts the generalizability of their outcomes [64,65,67].

We also found a significant, 13-year gap in research output on psychosocial interventions for patients with psychosis and schizophrenia. The paucity of publications highlights a significant “academic lag” often observed in rapidly developing economies, where research output in the social sciences frequently trails behind economic prosperity by several decades [73]. Although this publication gap cannot be explained definitively, several speculative contextual factors may offer possible explanations for this period. For example, the regional research landscape was dominated by foundational epidemiology and psychometric mapping rather than interventional trials between 2008 and 2021. Historically, mental health research in the GCC constituted less than 1% of total biomedical output, with nearly half of these studies being purely descriptive [39,74]. This period was also characterized by the gradual maturation of necessary legal and strategic frameworks, such as the implementation of Saudi Arabia’s Mental Health Care Law and Kuwait’s 2016 strategic consultation with the WHO, which were essential precursors to the shift toward community-based psychosocial care [39,58]. Furthermore, limited research resources were often redirected toward addressing regional crises and political unrest, resulting in a thematic focus on trauma, anxiety, and youth mental health rather than chronic psychotic disorders [75,76]. Consequently, the “silence” in the literature reflects a field that was still establishing its institutional infrastructure and prioritizing the mapping of prevalence before moving toward the systematic evaluation of psychosocial treatments.

The studies uncovered in this search depicted an overall disconnect between the academic recognition of the unique needs of individuals within the GCC and its systematic translation into intervention design. For example, the common use of spiritual attributions (e.g., jinn or witchcraft) and the preference for traditional healers [33,40], which can lead to delayed treatment and a poor prognosis [49,50,77], are not addressed in the included studies. Instead, the included interventions were almost entirely situated within biomedical or hospital-based frameworks. Family systems also represent an underdeveloped area; while caregiver interventions exist, they often focus on individual burden rather than integrating the collective family decision-making structures central to the region [44,70]. Indeed, studies have found great receptivity and response to interventions that integrate spiritually oriented beliefs or Islamic-oriented philosophies within interventions for schizophrenia [78,79]. Furthermore, studies that emphasize the idiosyncratic nature that family units as part of a larger culture play within the role of recovery for clients have shown promising results for minority communities [80,81,82]. Future studies in the GCC could integrate psychoeducation on common symptom attributions and consider collaborative work with local healers or sheikhs when developing or implementing a culturally adapted intervention for patients with psychosis or schizophrenia.

Anecdotally, many conversations observed locally have highlighted the ongoing need to address stigma within the public. This sentiment is often echoed in educational, clinical, and social media settings and is often reported as a primary barrier for patients with schizophrenia in the GCC [44]. It was therefore notable to see the limited number of studies that directly addressed this topic within the interventions reported. While one study discussed social exclusion as a contextual concern, the intervention itself focused on social phobia rather than evaluating stigma reduction [69]. Stigma in the GCC is deeply tied to family reputation and collective shame, which frequently leads to the avoidance of formal mental health services [52]. In Saudi Arabia, for instance, the shame associated with a diagnosis is associated with diminished quality of life, creating barriers to securing employment or pursuing marriage [47,83]. High levels of internalized stigma have also been documented in Qatar, where self-stigma correlates significantly with social support levels [72]. These findings suggest that future treatments cannot continue to address stigma solely as a secondary symptom but must prioritize multi-level designs that address it at the family and community levels [47,52,72]. Incorporating modules or exercises from stigma-specific protocols for patients with schizophrenia in a culturally relevant manner, such as that of the Be Outspoken and Overcome Stigmatizing Thoughts (BOOST) program [84], could be beneficial for patients and families in the region.

### 4.1. Limitations

Several methodological limitations should be considered when interpreting the findings of this review. First, although the search included sources in both English and Arabic, Arabic database interfaces varied in their support for advanced and reproducible search functions. This variability may have affected the systematic identification and documentation of all relevant Arabic literature. Second, gray literature was only partially captured. One doctoral dissertation was included, but dissertation-specific databases and other gray-literature sources were not searched systematically, which may have resulted in missed unpublished or non-indexed studies. Third, full data extraction was conducted by a single reviewer, and inter-rater consistency at the title and abstract stage was assessed using a 15% sample rather than full double-screening of all records. This approach may have introduced bias in study selection or data charting. To mitigate this risk, full-text screening disagreements were resolved through discussion, and an adjudicator was available for unresolved conflicts. Fourth, Arabic searching and extraction were conducted by an Arabic-fluent reviewer and translator, but a second independent reviewer was not available to verify all Arabic records. Fifth, publication bias remains possible, particularly because intervention studies with positive findings may be more likely to appear in published sources. Finally, although the intervention categories were developed through charting and discussion among the authors, the small number and heterogeneity of included studies mean that some degree of category bias is possible. Future reviews with larger evidence bases could apply more formal analytic approaches and conduct formal critical appraisals to refine these categories further.

### 4.2. Future Directions

Despite the limitations of the current regional evidence base, promising signs of progress are emerging. Two recently published protocols indicate a vital shift toward more systematic and scientifically robust research, focusing on culturally adapted family interventions in Oman and cognitive behavioral therapy adaptations in Saudi Arabia [47,85].

Building on this momentum, and to advance the field beyond its current methodological limitations, future research should establish GCC-wide collaborative networks for trial conduct, building upon the foundational protocols recently published by Aljuhani and Al Sawafi [44,47]. To improve transparency, researchers should continue to pre-register all study protocols in registries like OSF, ClinicalTrials.gov, or PROSPERO. A primary research priority should be the development of a shared set of core outcomes to ensure studies can be meaningfully compared across different Gulf settings.

These core outcomes should encompass clinical symptoms, functioning, and caregiver burden through the use of measures validated in Arabic or culturally adapted for Gulf populations that capture recovery concerns specific to the Gulf context, including family reputation, religious or supernatural illness attributions, pathways to care, marriage and employment prospects, and social reintegration [52]. Most critically, future designs must explicitly integrate stigma and self-stigma reduction components to address these pervasive barriers to well-being that remain largely unaddressed in the current regional literature [52]. Furthermore, future research requires greater consistency in reporting diagnostic classifications, intervention content, and follow-up timepoints. Any new intervention work should employ documented cultural adaptation frameworks, such as ADAPT-ITT or FRAME, and maintain rigorous reporting on adaptation fidelity. Geographically, targeted intervention research is needed in Bahrain and Qatar, where no eligible studies were identified despite documented psychosocial needs and high levels of internalized stigma [72].

## 5. Conclusions

This scoping review set out to map the translation process of psychosocial interventions for psychosis across Gulf countries, motivated by a growing body of evidence highlighting unique regional symptom presentations and urgent calls for culturally relevant care [40,44]. Despite these established needs, our findings reveal a surprising paucity of research output during the last decade, which urges caution when interpreting the current findings. At present, the existing evidence remains too limited and methodologically inconsistent to support strong practice or policy recommendations; however, improving methodological rigor, reporting clarity, and collaboration in intervention design and dissemination will be essential for generating actionable evidence in the future.

We recommend that future researchers adopt standardized concepts and validated evaluation tools to ensure cross-study comparability. Psychosocial interventions evaluated should include clearly documented cultural adaptation processes that explicitly address local symptom attributions, such as religious or spiritual interpretations, optional collaborations with traditional healers to bridge pathways to care [33,34,61], the formal involvement of caregivers, and targeted interventions addressing stigma [47,52,72]. These inclusions would help foster more culturally relevant interventions and potentially greater engagement with the larger healthcare systems that are present for patients with psychosis and schizophrenia.

## Figures and Tables

**Table 1:** Concepts and Search Terms.

Concept	English Search Terms	Arabic Search Terms
Psychosis or schizophrenia	psychosis OR psychotic OR schizophrenia OR schizophreni* OR “schizophrenia spectrum” OR “severe mental illness” OR “first episode psychosis”	الذهان OR ذهاني OR الفصام OR انفصام الشخصية OR طيف الفصام OR الاضطرابات الذهانية OR المرض النفسي الشديد
Psychosocial interventions	psychosocial OR psychotherapy OR therapy OR intervention* OR “cultural adaptation” OR cultural OR psychoeducation OR “family intervention” OR “family therapy” OR CBT OR “cognitive behavioral therapy” OR rehabilitation OR “community mental health” OR stigma OR “quality of life” OR recovery OR “functional recovery”	التدخل النفسي الاجتماعي OR التدخلات النفسية الاجتماعية OR العلاج النفسي OR العلاج المعرفي السلوكي OR العلاج السلوكي المعرفي OR التثقيف النفسي OR التدخل الأسري OR العلاج الأسري OR إعادة التأهيل OR التأهيل النفسي الاجتماعي OR الصحة النفسية المجتمعية OR الوصمة OR جودة الحياة OR التعافي OR التعافي الوظيفي
Gulf countries	“Saudi Arabia” OR KSA OR Kuwait OR Bahrain OR Qatar OR “United Arab Emirates” OR UAE OR Oman	السعودية OR المملكة العربية السعودية OR الكويت OR البحرين OR قطر OR الإمارات OR الإمارات العربية المتحدة OR عُمان OR عمان OR الخليج OR دول الخليج OR مجلس التعاون الخليجي

Note: The asterisk(*) indicates truncation where supported by the database syntax. All three concepts were combined using AND; terms within each concept were combined using OR. English terms were adapted across APA PsycINFO, PubMed, Embase, Scopus, and Web of Science using database-specific syntax. Arabic terms were used for targeted searching in Dar Almandumah, Al Manhal, and e-Marefa. Arabic database interfaces did not consistently support reproducible Boolean search strings; therefore, Arabic-language records were documented as additional sources and screened using the same eligibility criteria.

**Table 2:** Characteristics of Included Studies.

Study	Country	Intervention Category	Design	Sample and Diagnosis	Setting/Recipient
Alyamy (1995) [62]	Saudi Arabia	Transdiagnostic expressive therapy for inpatients	Exploratory doctoral dissertation case series	n = 3 adult male psychiatric inpatients; included one 35-year-old patient with paranoid schizophrenia	Inpatient
Wahass and Kent (1997) [61]	Saudi Arabia	Psychosocial intervention for voices	Small controlled intervention study	n = 6 male inpatients with ICD-10 schizophrenia and persistent auditory hallucinations; intervention n = 3, comparison n = 3	Inpatient
Alhamad et al. (2004) [63]	Saudi Arabia	Psychosocial rehabilitation platforms	Five-year prospective service evaluation	n = 354 engaged patients; transdiagnostic sample; schizophrenia was the largest diagnostic group, 27.4%	Day treatment
Al-Aradi and Farag (2002) [64]	Kuwait	Social and functional skills interventions	Controlled pre-post intervention study	n = 14 chronic schizophrenia inpatients; intervention n = 7, control n = 7	Inpatient
Al Sabwa and Abdelrahman (2008) [65]	Kuwait	Social and functional skills interventions	Controlled pre-post/follow-up intervention study	n = 16 chronic schizophrenia patients plus healthy comparison group	Psychiatric hospital, status not clearly specified
Al-Jadidi et al. (2021) [66]	Oman	Psychosocial rehabilitation platforms	Retrospective pre-post service evaluation	n = 54 community mental health service users; transdiagnostic sample; 61.1% diagnosed with schizophrenia	Community mental health service
Al-Rashoud (2021) [67]	Saudi Arabia	Caregiver-focused psychosocial interventions	Two-group pre-post intervention-control design with follow-up for intervention group	n = 40 mothers of people with schizophrenia; intervention n = 20, control n = 20	Caregiver
Elsabbahy et al. (2022) [68]	United Arab Emirates	Psychosocial rehabilitation platforms	Comparative day treatment/outpatient evaluation	Reported N = 123 with five non-completers; analyzed groups included 58 outpatients and 59 day treatment recipients; schizophrenia subgroup n = 43	Outpatient and day treatment
Al-Rashood (2025) [69]	Saudi Arabia	Social and functional skills interventions	One-group pre-post-follow-up intervention study	n = 11 recovered schizophrenia participants with elevated social phobia scores	Schizophrenia association/community organization
Sharif et al. (2025) [70]	Saudi Arabia	Caregiver-focused psychosocial interventions	Quasi-experimental intervention-control study	n = 60 family caregivers of people with schizophrenia; intervention n = 30, control n = 30	Caregiver

Note: n = sample size; N = total sample size.

**Table 3:** Intervention Characteristics and Delivery.

Study	Intervention and Delivery	Comparator	Cultural Adaptation or Local Context Reporting
Alyamy (1995) [62]	Six-week graphic reenactment art therapy structure: week 1 pre-testing, weeks 2 to 5 eight individual sessions, week 6 post-testing.	None	Explicitly situated art therapy within Saudi traditions, values, and Islam; no manualized religious adaptation process reported.
Wahass and Kent (1997) [61]	Culturally modified psychological intervention for persistent auditory hallucinations; three one-hour sessions per week over nine weeks, maximum 25 sessions.	Comparison group assessed over same period	Most explicit cultural adaptation; incorporated prayer, Quran reading, Islamic guidance audio, and doctrinal reframing of religious or superstitious voice content.
Alhamad et al. (2004) [63]	Psychiatric day treatment program including medication management, social support, rehabilitation, occupational individual and group sessions, physical training, computer classes/games, painting, handicrafts, needlework, and joinery.	No separate control group	Arabic translation/modification of outcome measure; no formal cultural adaptation framework reported.
Al-Aradi and Farag (2002) [64]	Token economy behavioral program plus medication; desired ward behaviors reinforced with tokens exchangeable for rewards.	Medication only	Arabic-language study in Kuwaiti inpatient context; locally adapted/study-specific measures; no formal cultural adaptation framework reported.
Al Sabwa and Abdelrahman (2008) [65]	CBT-based conversation skills program over four weeks; two sessions per week lasting 90 to 120 minutes.	Schizophrenia patient control group and healthy comparison group	Arabic-language study in Kuwaiti context; locally developed/adapted measure; no formal cultural adaptation framework reported.
Al-Jadidi et al. (2021) [66]	Community mental health service including outreach, crisis response, recovery-team input, rehabilitation, aftercare, assessment, medication provision/administration, case management, investigations, and multidisciplinary follow-up.	Pre-enrolment service period	Gulf community service context; no formal cultural adaptation process; multicomponent service makes psychosocial contribution difficult to isolate.
Al-Rashoud (2021) [67]	Researcher-developed CBT-based counseling program for caregivers; 14 sessions, three sessions per week.	Control group with no counseling program during study period	Arabic-language Saudi caregiver context; no specific religious intervention content or formal adaptation framework reported.
Elsabbahy et al. (2022) [68]	Psychosocial/community day treatment program compared with outpatient clinic care. Day treatment was described broadly without detailed session content, attendance frequency, or specific activities.	Outpatient clinic care	Measures translated/back-translated; one PCASEE item modified for comprehension; day treatment activities not detailed.
Al-Rashood (2025) [69]	Single-session group CBT intervention divided into stages; included cognitive restructuring, self-talk, social skills, assertiveness, modeling, public speaking, and role-play.	None	Arabic-language Saudi context; stigma/social exclusion discussed as context, but outcome was social phobia; no formal adaptation framework reported.
Sharif et al. (2025) [70]	Four-week telenursing psychoeducation program via Zoom; daily one-hour sessions using lectures, workshops, PowerPoint teaching, and weekly group discussions.	Routine care	Delivered in Saudi caregiver context; no religious adaptation described; FBIS had cited Arabic validity/reliability evidence.

Note: CBT = cognitive behavioral therapy; FBIS = Family Burden Interview Schedule; PCASEE = physical, cognitive, affective, social, economic, and ego functioning model.

**Table 4:** Outcome Measures and Key Findings.

Study	Outcomes/Measures	Key Findings	Schizophrenia-Specific Outcomes Reported?
Alyamy (1995) [62]	House-Tree-Person, Diagnostic Drawing Series, Brief Symptom Inventory, therapist observation, and qualitative drawing analysis.	For the schizophrenia case, some progress was described during sessions, but gains were not sustained at post-testing after an external stressor and severe psychotic episode.	Partially; one schizophrenia case within a three-person transdiagnostic case series.
Wahass and Kent (1997) [61]	Structured Auditory Hallucinations Interview and repeated 10-cm visual analogue scales for voice dimensions.	Improvements reported for two of three treated participants; comparison group showed no meaningful change.	Yes.
Alhamad et al. (2004) [63]	Modified Arabic version of Morningside Rehabilitation Status Scale, assessed every six weeks.	Sixty-five percent of engaged patients improved moderately or markedly; high-functioning rating increased from 0.6% to 29.7%; significant shift in MRSS scores.	No; outcomes reported for the full engaged sample.
Al-Aradi and Farag (2002) [64]	Study-specific/adapted social-skill measures: self-assertion, communication, and self-care.	Experimental group improved across self-assertion, communication, and self-care; post-test scores favored token economy group.	Yes.
Al Sabwa and Abdelrahman (2008) [65]	Researcher-developed Conversation Skills Test with 36 items across initiating, maintaining, and ending conversation.	Intervention group improved from pre-test to post-test; gains broadly maintained at follow-up; remained below healthy comparison group.	Yes.
Al-Jadidi et al. (2021) [66]	Admissions/readmissions, length of stay, and cost per admission.	Admissions/readmissions and length of stay decreased significantly after enrolment; cost per admission decreased after enrolment.	No; outcomes reported for the transdiagnostic community mental health service sample.
Al-Rashoud (2021) [67]	Researcher-developed psychological hardiness scale: commitment, challenge, control, and total hardiness.	Significant post-intervention improvement favoring intervention group; no post-test to follow-up difference, suggesting maintenance.	Not applicable; caregivers sample.
Elsabbahy et al. (2022) [68]	PCASEE quality-of-life model; BPRS for schizophrenia subgroup; GAF for all participants.	Schizophrenia subgroup improved in cognitive problems, social dysfunction, GAF, and BPRS; other quality-of-life domains not significantly different.	Yes.
Al-Rashood (2025) [69]	Thirty-six-item social phobia scale.	Significant pre- to post-intervention reduction in social phobia; no significant post-to-follow-up change.	Yes; sample was recovered schizophrenia participants with elevated social phobia.
Sharif et al. (2025) [70]	Family Burden Interview Schedule: financial burden, routine, leisure, family interaction, physical health, mental health, overall burden.	Post-intervention caregiver burden significantly lower across all domains; intervention-group mean total burden decreased from 2.46 to 1.49.	Not applicable; caregiver sample.

Note: BPRS = Brief Psychiatric Rating Scale; GAF = Global Assessment of Functioning; MRSS = Morningside Rehabilitation Status Scale; PCASEE = physical, cognitive, affective, social, economic, and ego functioning model.

## Data Availability

No new data were created or analyzed in this study. Data sharing is not applicable to this article.

## Supplementary Materials

The following supporting information can be downloaded at: https://www.mdpi.com/article/10.3390/jcm15135103/s1, File S1: Scoping Review Protocol; File S2: Search Strategies for English-Language Databases.

## Abbreviations

GCC	The Gulf Cooperation Council
WHO	World Health Organization
FRAME	Framework for Reporting Adaptations and Modifications-Enhanced
ADAPT-ITT	Assessment, Decision, Adaptation, Production, Topical experts-integration, Training, and Testing
