# Supplementary material for: Mapping Psychosocial Interventions for Psychosis and Schizophrenia Across Gulf Countries: A Scoping and Narrative Review
Source: J Clin Med. 2026 Jun 30;15(13):5103. doi: 10.3390/jcm15135103 (PMC13363634; doi:10.3390/jcm15135103)
Supplement: Supplementary file 1 [file jcm-15-05103-s001.zip › Supplementary File S1.pdf]

Supplementary File S1. Scoping Review Protocol

Title
Mapping Psychosocial Interventions for Schizophrenia and Psychosis Across Gulf Countries: A Scoping and Narrative Review

Review objective
The objective of this scoping review is to map the available evidence on psychosocial interventions for schizophrenia and psychosis evaluated in Gulf countries, and to describe the extent to which cultural adaptation is reported in these studies.

Review question
What psychosocial interventions for schizophrenia and psychosis have been evaluated in Gulf countries, and to what extent have cultural adaptations been reported?

Methodological approach
This scoping review will be guided by JBI methodology for scoping reviews and reported in accordance with PRISMA-ScR guidance.

Eligibility criteria
Studies will be eligible for inclusion if they evaluate a non-pharmacological psychosocial intervention or service model delivered to people with schizophrenia or psychosis, or to their family members or caregivers; are conducted in a Gulf country; and report an evaluation component with outcomes. Gulf countries are defined as Bahrain, Kuwait, Oman, Qatar, Saudi Arabia, and the United Arab Emirates.

Eligible outcomes will include, but will not be limited to, symptoms, relapse or readmission, functioning, quality of life, caregiver outcomes, social or functional skills, service use, and cost outcomes. Eligible study designs will include randomized and non-randomized controlled studies, quasi-experimental studies, pre–post evaluations, pilot studies, service or program evaluations, case reports, and case series when a psychosocial intervention is delivered and outcomes are described.

Medication-only studies, purely descriptive or observational studies without an intervention evaluation component, scale development or validation studies, and qualitative-only studies without an intervention evaluation component will be excluded. English- and Arabic-language publications will be eligible. Peer-reviewed studies and eligible grey literature will be considered when a full text is available. No date limits will be applied.

Information sources
English-language bibliographic databases will include APA PsycINFO, PubMed, Embase, Scopus, and Web of Science. Arabic-language sources will include Dar Almandumah, Al

Manhal, and e-Marefa. Additional relevant Arabic-language records identified through targeted searching will be screened using the same eligibility criteria as English-language records.

Search strategy
Searches will combine three core concepts: schizophrenia or psychosis, psychosocial interventions, and Gulf countries. English-language search terms will include terms such as psychosis, psychotic, schizophrenia, schizophreni*, schizophrenia spectrum, severe mental illness, first episode psychosis, psychosocial, psychotherapy, therapy, intervention*, cultural adaptation, psychoeducation, family intervention, CBT, rehabilitation, community mental health, stigma, quality of life, recovery, Saudi Arabia, Kuwait, Bahrain, Qatar, United Arab Emirates, UAE, Oman, and related terms.

Arabic-language searches will use equivalent Arabic terms for schizophrenia or psychosis, psychosocial intervention concepts, and Gulf countries. Because search functions may vary across Arabic database interfaces, Arabic-language records will be documented as additional sources rather than as fully reproducible database searches; however, all Arabic-language records identified through these sources will be screened using the same predefined eligibility criteria.

Study selection
All records will be imported into Rayyan for management and deduplication. Title and abstract screening will be conducted using predefined eligibility criteria. A second reviewer will independently screen a 15% sample of title and abstract records in blind mode to assess screening consistency. Full-text screening will be conducted independently by two reviewers, and disagreements will be resolved through discussion among the research team. An adjudicator will be available for unresolved disagreements.

Data charting
Data will be charted using a structured extraction template. Extracted items will include citation details, country, setting, study aim, study design, sample and diagnosis, intervention components, delivery characteristics, comparator, outcomes and measures, timepoints, key findings, cultural adaptation reporting, and study limitations. Arabic-language full texts will be screened and charted by an Arabic-fluent reviewer using the same framework as English-language studies.

Synthesis approach
Findings will be synthesized descriptively and narratively. Studies will first be charted by country, setting, study design, sample and diagnosis, intervention characteristics, comparator, outcomes, key findings, and reporting of cultural adaptation. Studies will then be grouped into intervention categories according to intervention target, delivery setting, and primary outcome domain. These categories will be discussed among the authors and refined until consensus is reached. The narrative synthesis will be organized around these intervention categories and interpreted in relation to cultural adaptation, methodological rigor, and gaps in the regional evidence base.

Critical appraisal
Consistent with the aims of this scoping review to map the literature rather than synthesize effectiveness or determine certainty, a formal critical appraisal was not required.

Protocol registration and availability
This protocol was not registered in a public registry. It is provided as Supplementary File 1 to support transparency regarding the review question, eligibility criteria, information sources, search strategy, study selection process, data charting approach, and synthesis methods.
