# Supplementary material for: Mapping Psychosocial Interventions for Psychosis and Schizophrenia Across Gulf Countries: A Scoping and Narrative Review
Source: J Clin Med. 2026 Jun 30;15(13):5103. doi: 10.3390/jcm15135103 (PMC13363634; doi:10.3390/jcm15135103)
Supplement: Supplementary file 1 [file jcm-15-05103-s001.zip › Supplementary File S2.pdf]

Supplementary File S2. Search Strategies for English-Language Databases

Table S1. Search terms used in the main review for English-language databases

Note. All searches were conducted on January 26, 2026. Records retrieved: PsycINFO, n = 80; PubMed, n = 37; Embase, n = 620; Scopus, n = 88; Web of Science Core Collection, n = 59. Final searches combined the numbered search components using AND.

Search terms	PsycINFO	PubMed	Embase	Scopus	Web of Science
**1. Psychosis**	Field: Abstract<br><br>psychosis OR psychotic OR schizophrenia OR schizophreni* OR "schizophrenia spectrum" OR "severe mental illness" OR "first episode psychosis"	("Psychotic Disorders"[MeSH] OR "Schizophrenia"[MeSH]<br>OR psychosis[tiab] OR psychotic[tiab] OR schizophrenia[tiab]<br>OR schizophreni*[tiab] OR "schizophrenia spectrum"[tiab]<br>OR "severe mental illness"[tiab]<br>OR "first episode psychosis"[tiab])	('psychosis'/exp OR 'schizophrenia'/exp OR 'psychotic disorder'/exp<br>OR psychosis:ti,ab OR psychotic:ti,ab OR schizophrenia:ti,ab<br>OR schizophreni*:ti,ab OR 'schizophrenia spectrum':ti,ab<br>OR 'severe mental illness':ti,ab<br>OR 'first episode psychosis':ti,ab<br>)	TITLE-ABS-KEY (psychosis OR psychotic OR schizophrenia OR schizophreni*<br>OR "schizophrenia spectrum" OR "severe mental illness"<br>OR "first episode psychosis")	TS = (psychosis OR psychotic OR schizophrenia OR schizophreni*<br>OR "schizophrenia spectrum" OR "severe mental illness"<br>OR "first episode psychosis")
**2. Intervention**	Field: Abstract<br><br>(<br>psychosocial OR psychotherapy OR psychoeducation OR CBT OR "cognitive behavioral therapy"<br>OR "family intervention" OR "family therapy" OR rehabilitation OR "community mental health"<br>OR "social skills	(<br>(<br>psychosocial[tiab] OR psychotherapy[tiab] OR psychoeducation[tiab]<br>OR CBT[tiab] OR "cognitive behavioral therapy"[tiab]<br>OR "family intervention"[tiab] OR "family therapy"[tiab]<br>OR rehabilitation[tiab] OR "community mental health"[tiab]<br>OR "social skills	(<br>(<br>'psychosocial intervention'/exp OR 'psychotherapy'/exp OR 'psychoeducation'/exp<br>OR 'family therapy'/exp OR 'rehabilitation'/exp<br>OR psychosocial:ti,ab OR psychotherapy:ti,ab OR psychoeducation:ti,ab<br>OR CBT:ti,ab OR 'cognitive behavioral therapy':ti,ab	TITLE-ABS-KEY (<br>(<br>psychosocial OR psychotherapy OR psychoeducation<br>OR CBT OR "cognitive behavioral therapy"<br>OR "family intervention" OR "family therapy"<br>OR rehabilitation OR "community mental health"<br>OR "social skills	TS = (<br>psychosocial OR psychotherapy OR psychoeducation OR CBT<br>OR "cognitive behavioral therapy" OR "family intervention"<br>OR "family therapy" OR rehabilitation OR "community mental health"<br>OR "social skills training" OR "case management" OR

Search terms	PsycINFO	PubMed	Embase	Scopus	Web of Science
	training" OR "case management" OR "supported employment") OR ((therapy OR intervention* OR program* OR treatment*) AND ("cultural adaptation" OR cultural OR stigma OR "quality of life" OR recovery OR "functional recovery"))	training"[tiab] OR "case management"[tiab] OR "supported employment"[tiab]) OR ((therapy[tiab] OR intervention*[tiab] OR program*[tiab] OR treatment*[tiab]) AND ("cultural adaptation"[tiab] OR cultural[tiab] OR stigma[tiab] OR "quality of life"[tiab] OR recovery[tiab] OR "functional recovery"[tiab])))	OR 'family intervention*':ti,ab OR 'family therapy':ti,ab OR rehabilitation:ti,ab OR 'community mental health':ti,ab OR 'social skills training':ti,ab OR 'case management':ti,ab OR 'supported employment':ti,ab) OR ((therapy:ti,ab OR intervention*:ti,ab OR program*:ti,ab OR treatment*:ti,ab) AND ('cultural adaptation':ti,ab OR cultural:ti,ab OR stigma:ti,ab OR 'quality of life':ti,ab OR recovery:ti,ab OR 'functional recovery':ti,ab)))	training" OR "case management" OR "supported employment") OR ((therapy OR intervention* OR program* OR treatment*) AND ("cultural adaptation" OR cultural OR stigma OR "quality of life" OR recovery OR "functional recovery")))	"supported employment" OR ((therapy OR intervention* OR program* OR treatment*) AND ("cultural adaptation" OR cultural OR stigma OR "quality of life" OR recovery OR "functional recovery")))
**3. GCC countries**	Field: Any Field<br><br>"Saudi Arabia" OR KSA OR Kuwait OR Bahrain OR Qatar OR	("Saudi Arabia"[tiab] OR KSA[tiab] OR Kuwait[tiab] OR Qatar[tiab] OR Bahrain[tiab]	('Saudi Arabia'/exp OR Kuwait/exp OR Qatar/exp OR Bahrain/exp OR 'United Arab Emirates'/exp OR	TITLE-ABS-KEY ("Saudi Arabia" OR KSA OR Kuwait OR Qatar OR Bahrain OR "United Arab	TS = ("Saudi Arabia" OR KSA OR Kuwait OR Qatar OR Bahrain OR "United Arab Emirates" OR UAE OR

Search terms	PsycINFO	PubMed	Embase	Scopus	Web of Science
	"United Arab Emirates" OR UAE OR Oman	OR "United Arab Emirates"[tiab] OR UAE[tiab] OR Oman[tiab])	Oman/exp OR 'Saudi Arabia':ti,ab OR KSA:ti,ab OR Kuwait:ti,ab OR Qatar:ti,ab OR Bahrain:ti,ab OR 'United Arab Emirates':ti,ab OR UAE:ti,ab OR Oman:ti,ab)	Emirates" OR UAE OR Oman)	Oman)
**Final Search**	1 AND 2 AND 3	1 AND 2 AND 3	1 AND 2 AND 3	1 AND 2 AND 3	1 AND 2 AND 3
